# The prevalence and associated factors of early childhood caries in 3- to 5-year-old children in Shaanxi Province, China: a cross-sectional study

**DOI:** 10.3389/froh.2026.1722341

**Published:** 2026-04-07

**Authors:** Zhiping Hu, Lingxia Zeng, Lin Huang, Leiting Du, Yurong Zhang, Yulu Wang, Riham Awad

**Affiliations:** 1 Key Laboratory of Shaanxi Province for Craniofacial Precision Medicine Research, College of Stomatology, Xi’an Jiaotong University, Xi'an, Shaanxi, China; 2 Clinical Research Center of Shaanxi Province for Dental and Maxillofacial Diseases, College of Stomatology, Xi’an Jiaotong University, Xi'an, Shaanxi, China; 3 Department of Epidemiology and Biostatistics, School of Public Health, Xi’an Jiaotong University Health Science Center, Xi'an, China; 4 Center for Chronic Disease Control and Prevention, Global Health Institution, Xi’an Jiaotong University, Xi'an, China; 5 Key Laboratory for Disease Prevention and Control and Health Promotion of Shaanxi Province, Xi'an, China; 6 Nutrition and Food Safety Engineering Research Center of Shaanxi Province, Xi'an, Shaanxi, China

**Keywords:** dietary behavior, early childhood caries, prevalence, risk factors, tooth position

## Abstract

**Objective:**

To determine the prevalence of early childhood caries (ECC), identify high-risk tooth sites, and analyze region-specific and early-life risk factors among 3- to 5-year-old children in Shaanxi Province, China.

**Methods:**

A cross-sectional study was conducted using stratified cluster random sampling to select 571 children from northern, central, and southern Shaanxi. Clinical oral examinations were performed following World Health Organization (WHO) criteria. Primary caregivers completed questionnaires regarding sociodemographic characteristics, birth outcomes, feeding practices, and dietary behaviors. Logistic regression analysis was employed to identify factors associated with ECC prevalence.

**Results:**

The overall ECC prevalence was 66.9%, with significant regional disparities (Northern: 85.6% vs. Central: 52.7%, *P* < 0.001). Caries rates increased from age 3 (54.1%) to age 5 (77.7%). The most severely affected tooth positions were the mandibular second primary molars (teeth 75, 85). High-risk surfaces included the mesial surfaces of maxillary central incisors and the occlusal surfaces of mandibular second molars. Multivariable analysis identified older age, lower BMI, and frequent sweet consumption as key risk factors. Additionally, mixed feeding, preterm birth, and low birth weight were significantly associated with caries in specific high-risk tooth positions.

**Conclusion:**

ECC prevalence in Shaanxi Province is high, characterized by distinct regional variations and specific patterns of tooth susceptibility. Preventive strategies should address perinatal factors and dietary behaviors, with targeted interventions for high-risk mandibular molars and maxillary incisors initiated in early childhood.

## Introduction

Oral health is integral to overall well-being across the lifespan, with early dental care—particularly during critical developmental windows—playing a foundational role in lifelong health (1, 2). Dental caries, the world's most common chronic bacterial disease, poses serious risks not only to oral health but also to systemic health. According to the 2016 Global Burden of Disease study, untreated caries in permanent teeth ranked as the second most prevalent condition globally, while caries in primary teeth ranked fifth (3).

Early Childhood Caries (ECC)—defined as the presence of one or more decayed, missing, or filled tooth surfaces in a child under 71 months—represents a major public health challenge (4). Global epidemiological analyses reveal that this problem is particularly acute in Asia, where the prevalence of ECC in preschool children is 52%, markedly higher than the rates in the Americas (48%) and Europe (43%) (5). The situation in China is equally concerning: recent data show a rise in ECC prevalence from 53% (2000–2016) to 59.3% (2017–2022) among preschool children (6). Children aged 3–5 years are particularly vulnerable due to thin, less mineralized enamel, dietary habits high in sugar, and limited oral hygiene capacity (7). ECC can lead to premature tooth loss, impaired mastication, malocclusion, and negatively affect nutrition, facial development, and psychosocial well-being (8, 9).

Given these impacts, effective prevention through behavior and risk factor management is essential. Although several studies have explored ECC risk factors (10, 11), few have analyzed how these factors differentially impact specific tooth positions and surfaces. From a public health modeling perspective, ECC is not a uniform disease; susceptibility varies significantly across the dental arch due to distinct local microenvironments and exposure patterns (e.g., bottle-feeding primarily affecting maxillary incisors vs. masticatory habits affecting molars). Understanding these site-specific patterns is crucial for precision public health. For instance, identifying high risks in proximal surfaces vs. occlusal surfaces requires different intervention strategies (e.g., flossing instruction vs. pit and fissure sealants). Therefore, this study aimed to determine ECC prevalence, map the high-risk tooth sites and surfaces, and identify the associated demographic, perinatal, and behavioral factors among 3- to 5-year-old children in Shaanxi Province.

## Methods and materials

### Study design

This study is based on a cross-sectional survey of 3- to 5-year-old children, conducted as part of the national key population oral health monitoring project, with Shaanxi Province serving as one of the sampling sites.

### Ethical considerations and subject selection

This study was approved by the Ethics Committee of Stomatology Hospital of Xi'an Jiaotong University [No. XJKQ-LL (2021) NO. 056]. Written informed consent was obtained from the primary caregivers of all participating children. Participation was strictly voluntary, and caregivers were informed of their right to withdraw at any time without penalty. No incentives were provided to avoid coercion.

### Study subjects and sampling methods

The sample size was calculated using the formula: *n* = deff [u2α *p* (1 − *p*)/*δ*2], where deff is the design effect and *p* is the proportion of children with ECC, *α* is set to 0.05 u2α = 3. The estimated rate *p* was 62.5% for deciduous tooth decay in 3- to 5-year-old children, and the relative allowable error of the overall rate *p* was controlled at 15%, *δ* = 0. 094, sampling design efficiency is taken as deff = 1.5. According to the calculation, the required sample size per stratum was 164, and the total sample size for the three age groups was 492. Considering an anticipated 10% refusal ratethe sample size was expanded to 542. Finally 571 children were actually enrolled. A stratified cluster sampling method proportional to population size (PPS) was adopted. From September 2021 to March 2022, the study was conducted at three urban-based kindergartens selected from the southern, central, and northern regions of Shaanxi Province. From each kindergarten, a roughly equal number of preschool children aged 3–5 years were recruited.

### Questionnaires

The primary caregivers of preschool children at the kindergartens completed two questionnaires. The first, based on the Fourth National Oral Epidemiological Survey Standard, assessed oral health in 3- to 5-year-old children (12). This questionnaire addressed caregiver education levels, feeding practices, sugary food consumption, oral hygiene habits, healthcare-seeking behaviors, and knowledge and attitudes towards their children's oral health. The second questionnaire focused on birth conditions, including gestational age, delivery method, birth weight, and any neonatal diseases. The “primary caregiver” was defined as the individual primarily responsible for the child's daily care and diet.

### Clinical oral examinations

Two trained pediatric dentists performed oral examinations using periodontal probes and flat mirrors under natural light, employing both visual inspection and probing techniques to evaluate dental caries in 3- to 5-year-old children. An oral specialist nurse documented the findings on an oral examination form. Following the examination, a detailed report was sent to the child's primary caregiver, outlining the child's oral health status and recommending appropriate treatments. Children aged 3–5 years present at the kindergartens on the examination day were invited to participate. Exclusion criteria included: (1) children whose caregivers did not provide consent; (2) children who were absent on the day of the survey; (3) children with systemic diseases affecting oral health; and (4) uncooperative children who refused examination.

### Outcomes variables

According to the “Methods for Oral Health Survey of Diseases” (Fifth Edition) established by the World Health Organization (13) and the Fourth National Oral Health Epidemiological Survey Program (14), a tooth is considered affected by caries if it shows visible cavities or significant destruction under the enamel, or if a probe detects softness in the pit and fissure surfaces or on smooth tooth surfaces. Teeth that have been previously filled, regardless of whether secondary caries is present, are also classified as affected by caries.

The caries rate is determined based on the presence or absence of caries. The overall caries rate is calculated by dividing the number of children with caries (numerator) by the total number of children examined (denominator). For specific dental positions, the caries rate is calculated by dividing the number of caries observed at those positions (numerator) by the total number of children with teeth in those positions (denominator). Similarly, for specific dental surfaces, the caries rate is calculated by dividing the number of carious surfaces (numerator) by the total number of dental surfaces examined (denominator).

### Explanatory variables

#### Socio-demographic characteristics

The factors considered include the child's gender, age, caregiver, child's residential area, caregiver's education level, and infant feeding methods. The feeding methods are categorized as predominantly breastfeeding, mixed breastfeeding and formula feeding, or predominantly formula feeding.

#### Birth outcomes indicators

Delivery method (natural or other), birth length, birth weight (low birth weight <2,500 g, high birth weight >4,000 g), gestational age (preterm <37 weeks, post-term >42 weeks), and whether the child had any neonatal diseases.

### Children's BMI

The height (kg) and weight (m) of each child are measured, and the BMI value is calculated according to the formula BMI weight (kg)/[height (m)]^2^. According to the Growth standard for children under 7 years of age (WS/423-2022), it is divided into three categories: low, moderate and high, and the cut-off points of BMI value are 25%, 75% and 100%, less than 25% is low, and more than 75% is high.

### Oral health knowledge and behaviors

Maternal Oral Health Knowledge Accuracy: The correctness rate of maternal oral health knowledge is calculated by dividing the number of questions answered correctly (numerator) by the total number of oral health knowledge questions (denominator). The accuracy rate is then categorized into tertiles—low, medium, and high level.

Oral Hygiene Behavior: a principal component analysis method was employed to identify key behavioral factors from 6 related problems. The scores for these behavioral factors were then categorized into tertiles—low, medium, and high level—for follow-up analysis.

### Quality control

Surveyors received comprehensive training and were tested to ensure competency. Completed questionnaires were reviewed on-site for accuracy and then double-entered into the database to prevent errors. Two dental professionals underwent both theoretical and clinical training from a qualified examiner prior to conducting oral examinations. They performed a standard consistency test with the examiner, achieving Kappa values greater than 0.8, indicating high inter-rater reliability.

All oral examinations were carried out by these professionally trained dentists. Questionnaires were distributed by kindergarten teachers to parents during pick-up and drop-off times. Surveyors checked the completed questionnaires for accuracy before collection. To verify consistency, a random sample of 5% of participants was re-surveyed 1 week later at local kindergartens, with Kappa values for key variables again exceeding 0.8.

### Statistical analysis

Continuous variables were described using means and standard deviations, while categorical variables were summarized by counts and percentages. To compare caries rates among different characteristic groups, statistical tests were employed as follows: Student's *t*-test for normally distributed data, Mann–Whitney *U* test for skewed data, and Pearson's chi-square test for categorical variables. A radar chart was utilized to illustrate the characteristics of high-risk tooth sites. Principal component analysis was conducted to identify key features of oral health behavior. A binary logistic regression model was developed to pinpoint risk factors associated with caries, and the results were presented using a forest plot. All statistical analyses were performed using R 4.3.1, with a two-tailed *P* value of <0.05 considered statistically significant.

## Results

### Characteristics of study participants and overall caries prevalence

A total of 571 children were included (327 boys, 57.3%). The overall caries prevalence was 66.9%, with a mean dmft of 3.1. As shown in Table 1, caries prevalence differed significantly by age (*P* < 0.001) and region (*P* < 0.001). Children in the northern region exhibited the highest prevalence (85.6%), followed by the southern (61.7%) and central regions (52.7%). Prevalence increased progressively with age, rising from 54.1% at age 3 to 77.7% at age 5. Other demographic factors did not show statistically significant differences (Table 1). Two factors related to children's oral hygiene habits were identified by principal component analysis, named as: F1-Sugary Food Behavior and F2-Brushing Behavior according to factors loading. The factor loading matrix detailing the contributions of each variable to these components is provided in Supplementary Appendix 1.

**Table 1:** Sociodemographic characteristics of the participants (*n* = 571).

Factors	Number (%)	Number with caries (%)	*χ*^2^/*T*	*P*
Level of education of guardians *n* (%)			0.770	0.681
Junior high school and below	83 (14.5)	56 (67.5)		
Senior high school and middle special school	156 (27.3)	100 (64.1)		
College and above	332 (58.1)	226 (68.1)		
Region			45.383	<0.001
South	240 (42.0)	148 (61.7)^a^		
Middle	150 (26.3)	79 (52.7)^a^		
North	181 (31.7)	155 (85.6)^b^		
Sex			1.467	0.226
Male	327 (57.3)	226 (69.1)		
Female	244 (42.7)	156 (63.9)		
Age (years)			26.120	<0.001
3	209 (36.6)	113 (54.1)^b^		
4	196 (34.3)	140 (71.4)^a^		
5	166 (29.1)	129 (77.7)^a^		
Gestational weeks			3.133	0.209
Premature birth	33 (5.8)	18 (54.5)		
Full-term birth	517 (90.5)	348 (67.3)		
Post-term birth	21 (3.7)	16 (76.2)		
Mode of delivery			0.028	0.868
Natural birth	327 (57.4)	220 (67.3)		
Others	243 (42.6)	161 (66.3)		
Birthweight			0.065	0.968
Low	26 (4.6)	17 (65.4)		
Normal	525 (91.9)	352 (67.0)		
High	20 (3.5)	13 (65.0)		
Birth height mean (SD), cm	50.47 (2.15)	50.55 (2.25)	34,979^c^	0.493
Neonatal diseases			0.048	0.827
Yes	165 (28.9)	112 (67.9)		
No (ref)	406 (71.1)	270 (66.5)		
Mode of feeding *n* (%)			1.428	0.490
Mainly breast feeding	326 (57.1)	213 (65.3)		
Combine of breast and artificial feeding	109 (19.1)	78 (71.6)		
Mainly artificial feeding	136 (23.8)	91 (66.9)		
BMI, mean (SD), kg/m^2^			5.240	0.073
Relatively low	49 (8.6)	32 (65.3)		
Moderate	362 (63.4)	254 (70.2)		
Relatively high	160 (28.0)	96 (60.0)		
Health knowledge level of caregivers			1.788	0.409
Low	144 (25.2)	91 (63.2)		
Middle	152 (26.6)	100 (65.8)		
High	275 (48.2)	191 (69.5)		
Sugary food behavior*			4.313	0.116
Low	189 (33.1)	117 (61.9)		
Middle	193 (33.8)	129 (66.8)		
High	189 (33.1)	136 (72.0)		
Brushing behavior*			1.186	0.553
Low	188 (32.9)	129 (68.6)		
Middle	194 (34.0)	124 (63.9)		
High	189 (33.1)	129 (68.3)		

a vs b (*P* < 0.05); region: South vs. Middle (*P* = 0.08), South vs. North (*P* < 0.001), Middle vs. North (*P* < 0.001); age (years): 3 age vs. 4 age (*P* < 0.001), 3 age vs. 5 age (*P* < 0.001), 4 age vs. 5 age (*P* = 0.173).

^c^*T* value.

*The factor related to children's oral hygiene habits were identified by principal component analysis, named as: F1-sugary food behavior and F2-brushing behavior according to factors loading.

### High-risk tooth positions and surfaces of caries in the children's deciduous teeth

**Figure 1:**
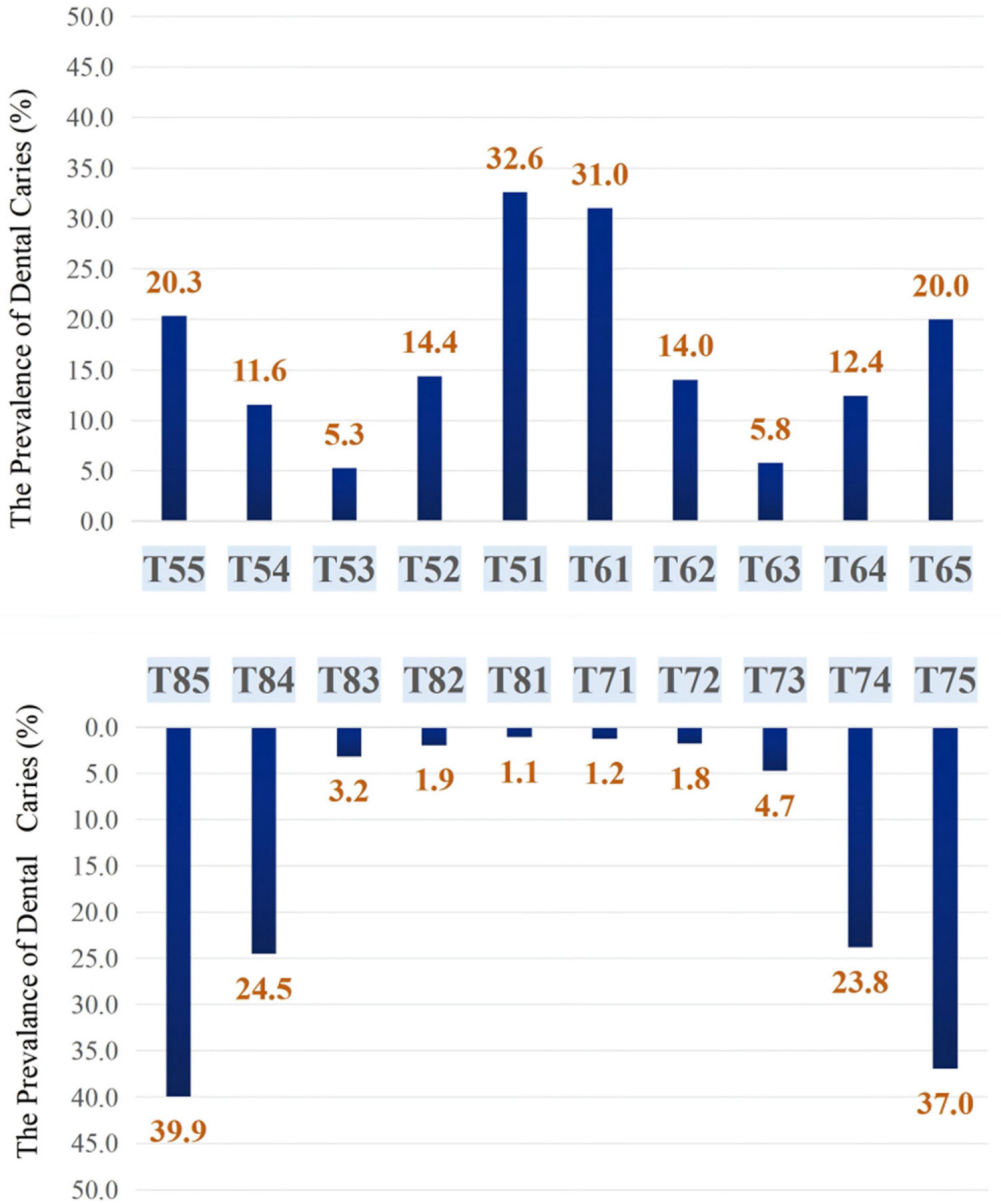
The prevalence of dental caries in primary teeth among children aged 3–5 years.

In all three age groups, caries were most common in teeth positions 75, 85, 51, 61, 74, and 84. The highest caries rates were found in mandibular teeth 85 (39.93%) and 75 (36.95%), followed by maxillary teeth 51 (32.57%) and 61 (31.00%). The lowest caries rates were observed in mandibular teeth 71 and 81 (Figure 1).

**Figure 2:**
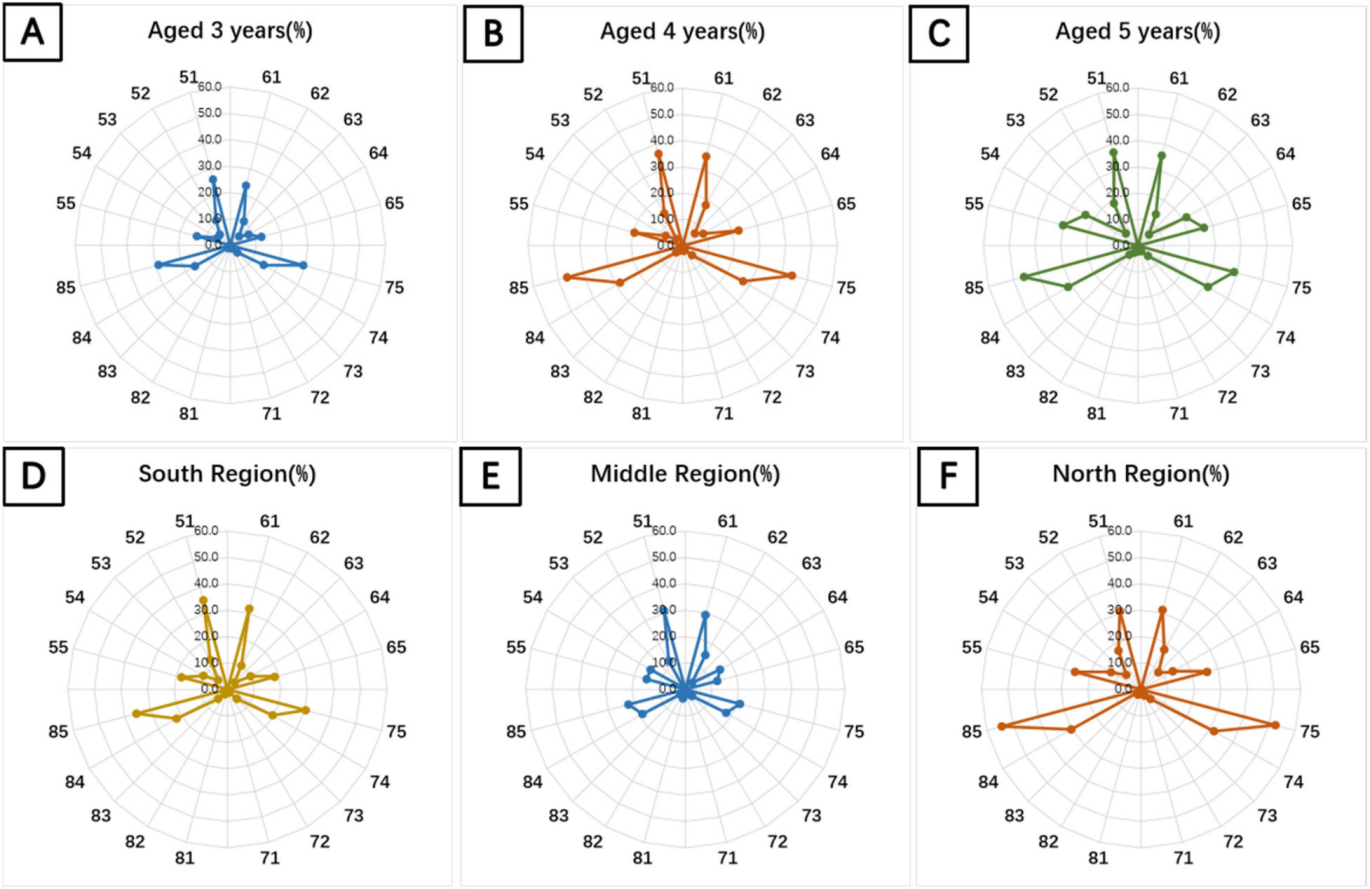
Depicts the caries prevalence across tooth positions in children stratified by age and region. Caries susceptibility increases with age, with significantly higher prevalence observed in the northern region compared to southern and central areas. **(A)** Age 3: Caries predominated in the mandibular teeth (75, 85, 74, 84) and maxillary incisors (51, 61), with a prevalence range of 23.44%–29.19%. **(B)** Age 4: Caries prevalence in susceptible dentition increased substantially to 35.20%–46.63%. **(C)** Age 5: Progressive prevalence noted (35.54%–45.78%), with emerging involvement of deciduous molars (55, 65, 54, 64). **(D)** Southern Region: Caries concentrated in mandibular posterior (75, 85, 74, 84) and maxillary anterior teeth (51, 61), prevalence 20.44%–34.17%. **(E)** Central Region: Demonstrated significantly lower caries rates across all susceptible positions. **(F)** Northern Region: Highest caries burden observed, particularly in teeth 75, 85, with expanded susceptibility patterns.

As age increase, the caries rate for the same tooth positions increased progressively. There was a rapid rise in caries rates from 3 to 4 years. The increase from 4 to 5 years was more gradual. The differences in caries rates across tooth positions were statistically significant (*P* < 0.05). Additionally, the number of high-risk tooth positions grew with age, and 5-year-olds exhibited higher caries prevalence specifically in positions 55 and 65. For detailed data are presented in Figures 2A–C.

There were regional differences in the distribution of high-risk tooth positions. In addition to positions 75, 85, 51, 61, 74, and 84, children in the northern region had a higher risk of caries in positions 55 and 65. Caries rates for the same tooth positions varied by region, with significantly higher rates in the northern region compared to the southern and central regions (*P* < 0.05). Shown on Figures 2D–F.

Different tooth surfaces also had varying caries prevalence. The highest caries rate was found on the mesial proximal surface of the upper right central incisor 51 at 26.27%, followed by the mesial proximal surface of the upper left central incisor 61 at 25.22%. For the lower right second primary molar 85, the occlusal surface had a 19.44% caries rate, and the occlusal surface of the lower left second primary molar 75 had a 17.55% caries rate (Supplementary Appendix 2).

### Factors influencing primary tooth caries

#### Multivariate analysis of factors associated with dental caries

**Figure 3:**
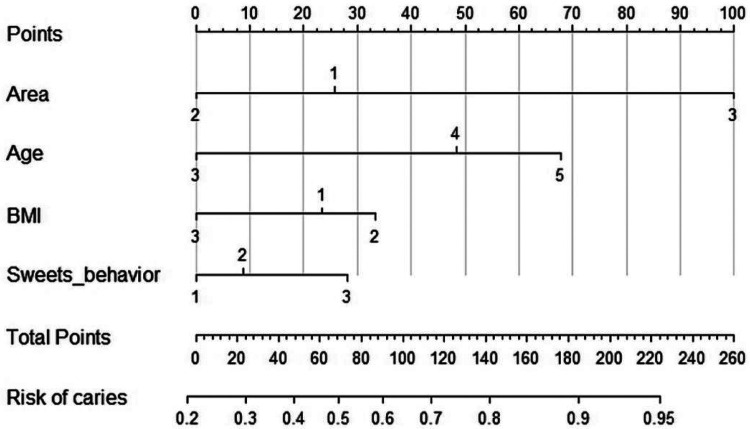
Nomogram for predicting dental caries risk in children multivariable logistic regression analysis identified that age, geographic region, BMI category, and sweet food consumption as significant determinants of dental caries in children. Multicollinearity was assessed using the variance inflation factor (VIF), with problematic collinearity defined as VIF > 5. The results showed that all variables had VIF values below 2, indicating no presence of multicollinearity. The Hosmer-Lemeshow test was used to evaluate the goodness-of-fit of the model, yielding a chi-square value of 8.070 and a *P*-value of 0.427, which suggests that the model fits the data well.

Age, region, BMI category, and frequency of sugary food intake were key factors influencing dental caries in children. The risk of caries increased with age. Children in the northern region experienced a significantly higher caries rate and had more high-risk tooth positions compared to those in the southern and central regions, with an adjusted odds ratio (AOR) of 7.50 (95% CI 4.17, 13.48) for the northern vs. central regions. Interestingly, children with higher BMI had a lower risk of caries. Conversely, a higher frequency of sugary food intake was linked to an increased risk of caries. For visual details, as shown on Figure 3.

**Figure 4:**
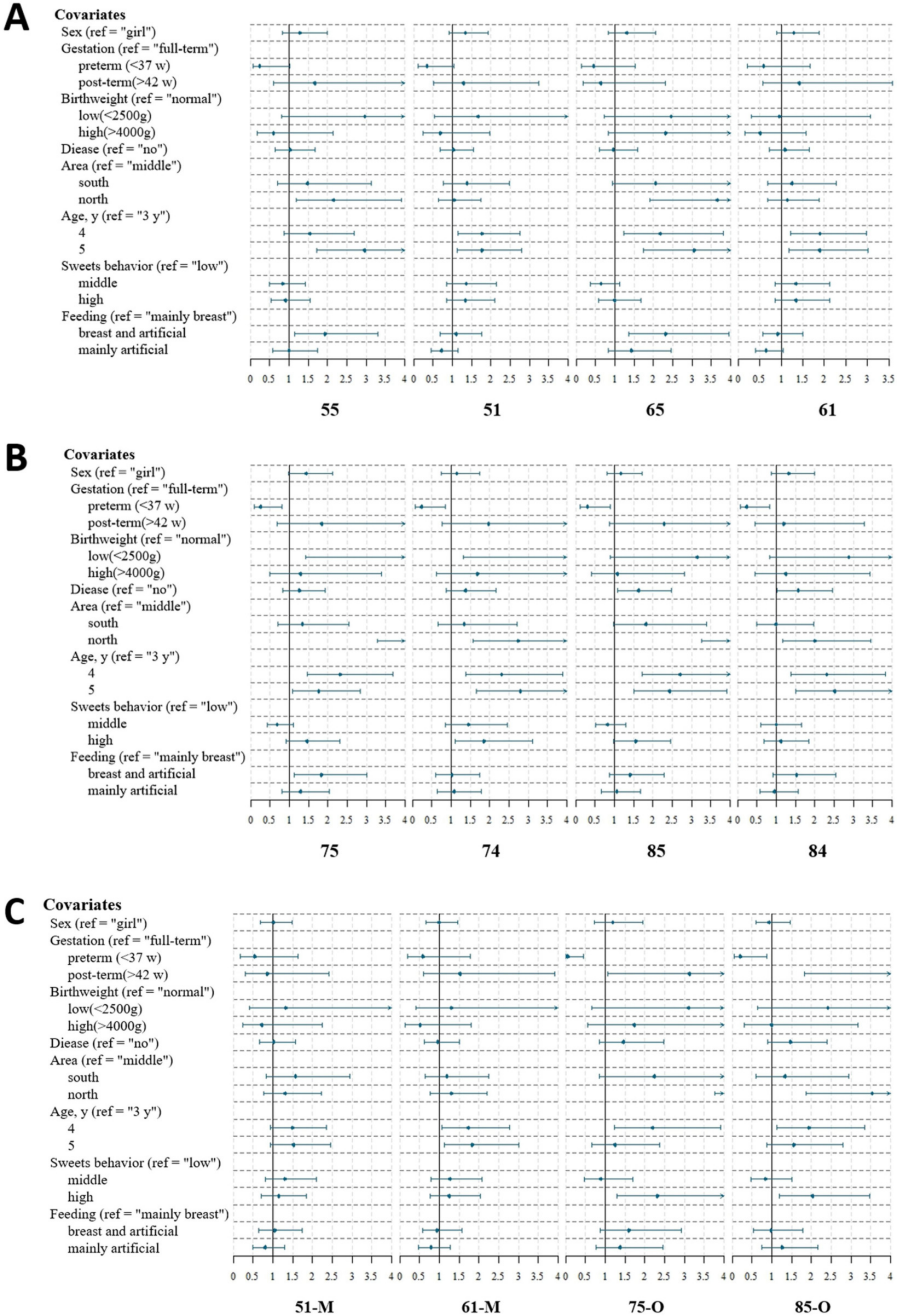
Analysis of influencing factors for caries in high-risk tooth sites and surfaces among children. This figure presents the analysis of factors influencing caries development in the most susceptible tooth sites and surfaces among children. **(A)** Factors associated with caries in the four high-risk maxillary tooth sites. **(B)** Factors associated with caries in the four high-risk mandibular tooth sites. **(C)** Factors associated with caries in the four high-risk tooth surfaces across maxillary and mandibular dentitions. O, occlusal surface; M, mesial proximal surface.

In this study, high-risk tooth positions for caries were identified as follows: in the upper primary teeth, positions 55, 65, 51, and 61; and in the lower primary teeth, positions 75, 85, 74, and 84. Besides the main influencing factors such as age, region, BMI, and sugary food behaviors, other factors also impacted caries risk in these specific tooth positions. Children with mixed feeding (both breast and formula) exhibited higher caries rates in the upper positions 55 and 65, as well as in the lower left positions 74 and 75. Premature infants (born before 37 weeks) had a lower risk of caries in high-risk positions of the lower jaw compared to full-term children. Conversely, children with low birth weight (<2,500 g) and those with neonatal diseases were at a higher risk of caries in high-risk positions of the lower jaw (Figures 4A,B).

High-risk tooth surfaces for caries were concentrated on the mesial proximal surfaces of upper central incisors (51, 61) and occlusal surfaces of lower second primary molars (75, 85). Besides age, region, and sugary food behaviors, prematurity and post-term birth were major factors influencing caries on specific tooth surfaces. Compared to full-term children, premature infants had a lower risk of caries on the occlusal surfaces of lower positions 75 and 85, while post-term children had a higher risk. In the northern region and among children with frequent sugary food intake, the risk of caries on the occlusal surfaces of the lower high-risk positions (75, 85) was higher. The risk of caries on the occlusal surfaces of lower high-risk positions (75, 85) increased with age, and the risk for the mesial proximal surface of the upper left central incisor (61) increased significantly (Figure 4C).

## Discussion

### Dental caries prevalence and regional differences

This cross-sectional study of children aged 3–5 years in Shaanxi Province revealed significant levels and pronounced spatial heterogeneity in the prevalence of early childhood caries (ECC). The overall ECC prevalence was 66.9%, substantially higher than the WHO global target of “ECC prevalence below 50% by 2020” (15) and also exceeded the average rate for Chinese children of the same age group (62.5%) (16), indicating a serious overall challenge to children's oral health in the region. Further cross-regional comparisons showed that the ECC prevalence in Shaanxi was comparable to that in some inland provinces (e.g., Shandong, Henan) (9, 17), but was markedly higher than that in eastern coastal areas (e.g., Shanghai, Zhejiang, 45%–55%) (13, 14, 18, 19) and developed Asian countries (e.g., Japan, South Korea, 50%–60%) (20), while remaining lower than that in several developing regions (e.g., India, Vietnam, 70%–80%) (21–23). This pattern suggests a potential coupling between caries prevalence and regional socioeconomic development.

Analysis by age revealed a clear increasing trend in caries prevalence from ages 3–5 (54.1% → 71.4% → 77.7%). The most rapid progression occurred between ages 3 and 4 (an increase of 32.0%), significantly exceeding the national average growth rate (25.2%) (24), highlighting this period as a critical window for primary prevention of ECC.

Notably, the distribution of ECC within Shaanxi exhibited significant gradients: the northern region had the highest prevalence (85.6%), while the central region had the lowest (52.7%). Children in the north faced an approximately 7.50-fold higher risk of ECC than those in the central region (*P* < 0.05). This internal disparity aligns with the macro-level trend observed nationally, where Northwest China has the highest caries rates and Central China the lowest, underscoring how structural factors—such as regional economic status, healthcare resource allocation, and dietary habits—collectively shape the geographical heterogeneity of caries prevalence (25–29).

### Influence of birth and nutritional factors

Birth conditions and early nutritional factors play a foundational role in tooth development and subsequent caries susceptibility (46). Our findings elucidate their systemic influence on early childhood caries (ECC).

Low birth weight (LBW, <2,500 g) was a significant risk factor (OR = 2.1), consistent with its established link to developmental defects of enamel (DDE). LBW is associated with reduced enamel thickness and mineralization, directly compromising the tooth's resistance to acid. This suggests that intrauterine nutritional deficiencies can irreversibly disrupt tooth germ differentiation and enamel matrix formation (30, 31).

Nutritionally, we observed an inverse association between higher BMI and caries risk (OR = 0.56), a correlation supported by previous studies (32–36). This apparent protective effect is likely not due to obesity *per se*, but a proxy for better overall nutrition. Two key mechanisms may explain this: first, diets richer in protein and fat often correspond to lower intake of free sugars; second, adequate energy and nutrient status support healthy salivary flow and buffering capacity, whereas undernourishment can lead to reduced salivary secretion and weakened oral clearance.

Feeding practices further modulated risk. Mixed feeding (breast milk combined with formula) significantly increased caries risk in primary molars (OR = 1.7). This may result from a synergistic cariogenic effect of lactose and sucrose, combined with prolonged oral retention during night-time feeding when salivary flow is reduced (37–40). Notably, the caries risk in our cohort was most pronounced in mandibular molars, contrasting with the anterior dominance of classic “nursing bottle caries” (9, 19, 41), indicating that feeding posture and local microenvironment critically influence caries localization.

### Behavioral determinants of early childhood caries

Our findings underscore critical behavioral determinants of early childhood caries (ECC), revealing a persistent disconnect between nominal adherence to oral hygiene and effective caries prevention. Although reported toothbrushing frequency in our cohort (≥twice/day: 33.1%) exceeded the national average (20.1%), the ECC prevalence in Shaanxi remained elevated (66.9% vs. 62.5% nationally), indicating that brushing frequency alone is an inadequate predictor of oral health outcomes.

The central issue lies in an effectiveness gap in oral hygiene practices. Despite reasonable brushing frequency, caregiver knowledge of proper technique was limited (correct knowledge rate: 48.2%), likely resulting in insufficient mechanical plaque removal—particularly from the pit-and-fissure surfaces of mandibular second primary molars, which emerged as high-risk sites in this study. Ineffective technique, coupled with low utilization of adjuvant measures such as dental floss and potentially suboptimal fluoride toothpaste use, fosters a microenvironment favorable to biofilm accumulation and caries initiation.

Dietary behaviors, especially sugar exposure patterns, further modulate caries risk. Frequent consumption of sugary foods and drinks—particularly those with high adhesiveness—prolongs the retention of fermentable carbohydrates on retentive occlusal surfaces. We identified a strong association between sugary snack intake and caries on mandibular primary molars 74, 75, 84, 85, suggesting that the frequency of sugar intake may be more critical than total quantity. Repeated acid challenges overwhelm salivary buffering capacity and impede enamel remineralization, thereby accelerating caries progression in susceptible sites (42–44).

Family context and parental practices serve as crucial mediators of behavioral risk (45). Low levels of accurate oral health knowledge among caregivers not only perpetuate ineffective brushing but also normalize frequent sugar consumption. Parental caries experience and the absence of regular dental visits further compound risk, shaping children's oral health behaviors and attitudes from an early age.

### Site-specific patterns and public health implications

Unlike previous studies that often focus solely on overall prevalence, our modeling of tooth-specific risks offers novel insights. We observed a high susceptibility in mandibular second molars (85, 75), particularly on occlusal surfaces. This pattern differs slightly from the classic “bottle-feeding” pattern which predominantly destroys maxillary incisors. This suggests that in this population, while bottle-feeding remains a risk, dietary retention of sticky carbohydrates (common in the local noodle-based diet) and lack of effective masticatory cleaning or pit-and-fissure sealing may play a larger role in driving posterior caries.

This finding challenges the “one-size-fits-all” prevention approach. For public health planning in Shaanxi, simply educating on bottle-feeding is insufficient. Resources should be reallocated towards promoting early flossing (for proximal surfaces of incisors) and, crucially, the widespread application of pit and fissure sealants for primary molars, which is often neglected compared to permanent molars.

## Limitation

This study is subject to several limitations. First, the sample was drawn exclusively from urban kindergartens within a single province, which may limit the generalizability of the findings to rural or socioeconomically diverse populations. Second, although key variables were considered, residual confounding may remain due to unmeasured or incompletely quantified factors such as detailed dietary habits and family socioeconomic status. Finally, the cross-sectional design precludes the establishment of causal relationships, highlighting the need for longitudinal or intervention studies in the future.

## Conclusion

The findings reveal a high prevalence of early childhood caries among preschool children in Shaanxi Province, with distinct patterns of tooth and surface susceptibility. Caries development was influenced by a range of factors, including geographic region, age, birth history, dietary habits, and nutritional status. These results underscore the need for a life-course approach to caries prevention, starting prenatally with maternal oral health education and extending through early childhood with structured dental assessments and home-based care guidance. Particular attention should be directed toward children in northern areas and those in older age groups. From a policy and clinical implication, it is essential to strengthen the integration of oral health into primary health care, reduce regional disparities in resources, and implement targeted, stepped-care interventions based on caries risk profiles and site-specific susceptibility.

## Data Availability

The raw data supporting the conclusions of this article will be made available by the authors, without undue reservation.
