# Supplementary material for: The prevalence and associated factors of early childhood caries in 3- to 5-year-old children in Shaanxi Province, China: a cross-sectional study
Source: Front Oral Health. 2026 Apr 7;7:1722341. doi: 10.3389/froh.2026.1722341 (PMC13095519; doi:10.3389/froh.2026.1722341)
Supplement: Supplementary file 1 [file Supplementaryfile1.docx]

**Appendix 1. Loading matrix of Factors influencing oral health behaviors**

| **Component Matrix** | **Component** | |
| --- | --- | --- |
|  | **F1-Sweets Behavior** | **F2-Brushing Behavior** |
| Consumption of desserts | **0.639** | -0.290 |
| Consumption of sweetened beverage | **0.708** | -0.314 |
| Consumption of sweetened dairy | **0.622** | -0.136 |
| Consumption of milk, beverage, or desserts after brushing teeth and before bedtime | **0.542** | 0.192 |
| Age of start brushing tooth | 0.208 | **0.737** |
| Frequency of daily toothbrushing | 0.318 | **0.739** |

Notes: F1 & F2 Explained 51.016% of variance (F1 explained 28.119% of Variance, F2 explained 22.897% of Variance) .
