# Supplementary material for: The prevalence and associated factors of early childhood caries in 3- to 5-year-old children in Shaanxi Province, China: a cross-sectional study
Source: Front Oral Health. 2026 Apr 7;7:1722341. doi: 10.3389/froh.2026.1722341 (PMC13095519; doi:10.3389/froh.2026.1722341)
Supplement: Supplementary file 2 [file Supplementaryfile2.pdf]

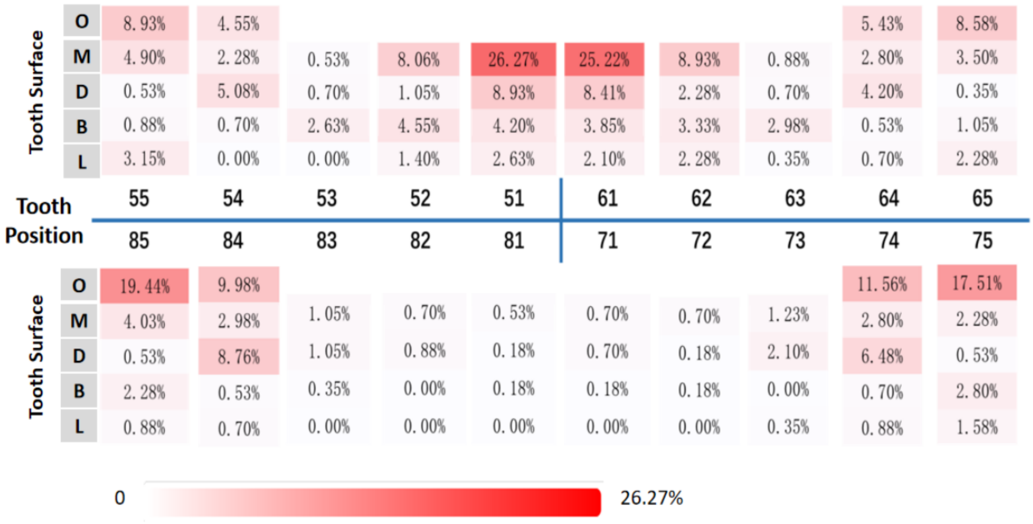

Appendix 2. Prevalence of Dental Caries on Different Tooth Surfaces in Children Aged 3-5 Years

Notes: O, Occlusal surface; M, Mesial proximal surface; D, Distal surface; B, Buccal surface; L, Lingual surface.
