# Supplementary material for: The prevalence and associated factors of early childhood caries in 3- to 5-year-old children in Shaanxi Province, China: a cross-sectional study
Source: Front Oral Health. 2026 Apr 7;7:1722341. doi: 10.3389/froh.2026.1722341 (PMC13095519; doi:10.3389/froh.2026.1722341)
Supplement: Supplementary file 3 [file Supplementaryfile3.pdf]

Survey on Oral Health Status of Children
(For parents/guardians of children aged 3–5 only)
Dear Parent/Guardian,
Hello! To better understand the oral health status of young children and promote their oral well-being, we sincerely invite you to participate in this questionnaire. It will take approximately 5–10 minutes to complete. Participation is voluntary and will not affect you in any way. We promise to keep all information strictly confidential. Thank you very much for your time and cooperation. Best wishes!
Respondent ID: □□□□□□□ (to be filled by staff)
Child's Name: __________
Date of Survey: ________ / ________ / ________
Note: Only the child's parents or grandparents may complete this questionnaire.

---

1. Questionnaire on Oral Health Status
Please answer truthfully based on your actual situation. Tick "√" in the box next to the most appropriate answer. Unless otherwise noted, only one answer should be selected for each question.

(1) You are the child's:
□ Father □ Mother □ Grandparent

(2) How was your child fed within the first six months after birth?
□ Exclusively breastfed
□ Primarily breastfed
□ Equally breastfed and formula-fed
□ Primarily formula-fed
□ Exclusively formula-fed

(3) How often does your child consume the following?

Food/Drink	≥2 times/day	1 time/day	2–6 times/week	1 time/week	1–3 times/month	Rarely/Never
Sweets (biscuits, cakes, candies, chocolate, etc.)	□	□	□	□	□	□
Sweetened beverages (sodas, juice drinks, lemonade, etc.)	□	□	□	□	□	□
Sweetened milk, yogurt, tea, soy milk,	□	□	□	□	□	□

Food/Drink	≥2 times/day	1 time/day	2–6 times/week	1 time/week	1–3 times/month	Rarely/Never
coffee, milk tea, etc.						

(4) Does your child have the habit of eating or drinking (milk, drinks, sweets) after brushing teeth at night?
□ Once daily □ 2–6 times/week □ Once/week □ 1–3 times/month □ Never

(5) At what age did your child begin brushing their teeth? (If “Never brushes” is selected, skip to Question 9)
□ 6–12 months □ 1–2 years □ 2–3 years □ 3–4 years
□ 4–5 years □ 5 years or older □ Never brushes

(6) How often does your child brush their teeth?
□ Twice or more daily □ Once daily □ Not every day

(7) How often do you assist your child with brushing?
□ 1–2 times/day □ 2–6 times/week □ Once/week
□ 1–3 times/month □ Never

(8) Does your child use fluoride toothpaste?
□ Yes □ No □ Don’t know □ Doesn’t use toothpaste

(9) How often do you help your child use dental floss?
□ Never □ Occasionally □ Weekly □ Daily

(10) Has your child ever visited a dentist?
□ Yes □ Never (If selected, skip to Question 14)

(11) What was the reason for your child's most recent dental visit? (multiple choices allowed)
□ Consultation/Advice □ Preventive care □ Emergency/pain
□ Non-emergency treatment □ Don’t know/Can’t remember

(12) Total dental expenditure in the past year: _____ yuan (If unknown or unwilling to answer, write “N”)

(13) Proportion of that cost you paid personally: _____% (If unknown or unwilling to answer, write “N”)

(14) How would you rate your child's oral health status?
□ Very good □ Good □ Fair □ Poor □ Very poor

(15) Do you think the following statements are correct?

Statement	Correct	Incorrect	Don’t know
Bleeding gums during brushing is normal	□	□	□
Bacteria can cause gingivitis	□	□	□
Brushing does not help prevent gum bleeding	□	□	□
Bacteria can cause dental caries	□	□	□
Eating sweets causes cavities	□	□	□
Decayed baby teeth don’t need treatment	□	□	□
Sealants can prevent children’s tooth decay	□	□	□
Fluoride is not useful for protecting teeth	□	□	□

(16) To what extent do you agree with the following statements?

Statement	Agree	Disagree	Neutral
Oral health is important for quality of life	□	□	□
Regular dental check-ups are necessary	□	□	□
Dental condition is mostly genetic	□	□	□
Prevention of dental disease depends on oneself	□	□	□
Protecting a child’s first permanent molars is crucial	□	□	□
A mother’s oral health affects her child’s teeth	□	□	□

(17) What is the highest level of education attained by the child’s mother?
□ Primary school or below □ Junior high □ High school/Technical secondary
□ College/University □ Master’s or above

(18) Number of people living in your household: _____ (Write “N” if unknown or unwilling to answer)

(19) Total household income: _____ 10,000 yuan/year (Write “N” if unknown or unwilling to

answer)

---

2. Child Birth and Early Health Status Questionnaire

(To be completed by the child's parents or grandparents only)

K1. Date of birth: 20____ / ____ / ____

K2. Gestational age at birth:

□ Premature (<37 weeks)

□ Full-term (37–42 weeks)

□ Post-term (>42 weeks)

K3. Mode of delivery:

□ Vaginal birth

□ Assisted delivery (breech/forceps/vacuum)

□ Cesarean section

□ Other: ___________

□ Unknown

K4. Birth weight:

□ <2500g □ 2500–4000g □ >4000g

K5. Birth length: _____ cm

K6. Diagnosed with any of the following conditions during the neonatal period? (Multiple choices allowed)

□ Pathological jaundice □ Pneumonia □ Hypoxic-ischemic encephalopathy

□ Asphyxia □ Intracranial hemorrhage □ Hematologic disorder

□ Acute respiratory distress syndrome □ Birth trauma □ Sepsis

□ Tetanus □ Umbilical infection □ Meconium aspiration syndrome □ Other: ___________

K7. Current height: _______ cm

K8. Current weight: _______ kg

K9. Date of measurement: 20____ / ____ / ____

K10. Does the child currently use toothpaste containing probiotics?

□ Yes □ No □ Don't know

K11. Type of toothpaste currently used:

□ Children's toothpaste □ Adult toothpaste □ Don't know
